# Comparative biochemical analysis of three members of the *Schistosoma mansoni* TAL family: Differences in ion and drug binding properties

**DOI:** 10.1016/j.biochi.2014.10.015

**Published:** 2015-01

**Authors:** Charlotte M. Thomas, Colin M. Fitzsimmons, David W. Dunne, David J. Timson

**Affiliations:** a School of Biological Sciences, Queen's University Belfast, Medical Biology Centre, 97 Lisburn Road, Belfast, BT9 7BL, UK; b Institute for Global Food Security, Queen's University Belfast, 18-30 Malone Road, Belfast, BT9 5BN, UK; c Department of Pathology, University of Cambridge, Cambridge, CB2 1QP, UK

**Keywords:** Schistosomiasis, Calcium binding protein, Tegumental allergen, Praziquantel, EF-hand protein

## Abstract

The tegumental allergen-like (TAL) proteins from *Schistosoma mansoni* are part of a family of calcium binding proteins found only in parasitic flatworms. These proteins have attracted interest as potential drug or vaccine targets, yet comparatively little is known about their biochemistry. Here, we compared the biochemical properties of three members of this family: SmTAL1 (Sm22.6), SmTAL2 (Sm21.7) and SmTAL3 (Sm20.8). Molecular modelling suggested that, despite similarities in domain organisation, there are differences in the three proteins’ structures. SmTAL1 was predicted to have two functional calcium binding sites and SmTAL2 was predicted to have one. Despite the presence of two EF-hand-like structures in SmTAL3, neither was predicted to be functional. These predictions were confirmed by native gel electrophoresis, intrinsic fluorescence and differential scanning fluorimetry: both SmTAL1 and SmTAL2 are able to bind calcium ions reversibly, but SmTAL3 is not. SmTAL1 is also able to interact with manganese, strontium, iron(II) and nickel ions. SmTAL2 has a different ion binding profile interacting with cadmium, manganese, magnesium, strontium and barium ions in addition to calcium. All three proteins form dimers and, in contrast to some *Fasciola hepatica* proteins from the same family; dimerization is not affected by calcium ions. SmTAL1 interacts with the anti-schistosomal drug praziquantel and the calmodulin antagonists trifluoperazine, chlorpromazine and W7. SmTAL2 interacts only with W7. SmTAL3 interacts with the aforementioned calmodulin antagonists and thiamylal, but not praziquantel. Overall, these data suggest that the proteins have different biochemical properties and thus, most likely, different *in vivo* functions.

## Highlights

• SmTAL1, SmTAL2 and SmTAL3 have different predicted structures.
• SmTAL1 and SmTAL2, but not SmTAL3 reversibly bind calcium ions.
• SmTAL1 and SmTAL2 bind an overlapping but different range of other cations.
• All three proteins form dimers; calcium ions have no effect on the dimerization.
• SmTAL1 binds to praziquantel; SmTAL1 and SmTAL3 bind calmodulin antagonists.

## Graphical abstract

**Figure:**
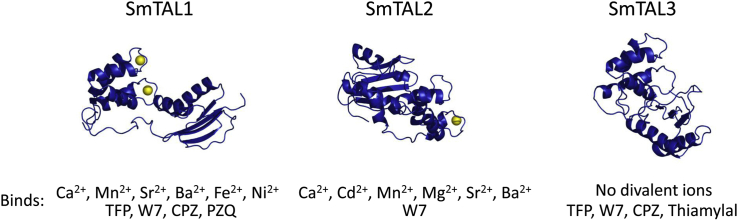

## 1 Introduction

Schistosomiasis (or bilharzia) is the most common cause of death from a parasitic disease after malaria [1,2]. Infection with blood flukes from the genus *Schistosoma* affects approximately 230 million people, primarily in tropical regions [3,4]. The disease can be treated effectively with the drug praziquantel (PZQ) [5]. The mechanism of action of this drug is currently unknown although evidence suggests that it acts to disrupt calcium signalling processes in the fluke, possibly through the antagonism of voltage-gated ion channels [6,7]. Alternative mechanisms of action which have been proposed include antagonism of adenosine uptake and interference with the function of myosin regulatory light chains [8,9]. Although resistance to PZQ has been generated under laboratory conditions, there are as yet no definitive reports of the emergence of resistance in clinical conditions [10,11]. Resistance to oxamniquine, an alternative drug for the treatment of *Schistosoma mansoni* infections, has been reported [12]. Other helminth parasites have also demonstrated the ability to evolve resistance to commonly used drugs. For example, there are now numerous reports that liver fluke *Fasciola hepatica* can become resistant to triclabendazole and various species of intestinal nematodes have developed resistance to ivermectin [13,14]. Thus, it seems likely that clinically significant resistance to PZQ will, eventually, appear.

Calcium signalling is a key process in all eukaryotic cells [15]. These signalling processes are mediated by calcium binding proteins, of which the best characterised is calmodulin [16]. Typically they coordinate calcium ions using one, or more, EF-hand motifs [17]. Following binding, the proteins often undergo conformational changes which alter their interactions with other molecules. In parasitic platyhelminthes (flatworms) there is an unusual family of calcium binding proteins which have not been found in any other group of organisms. These proteins consist of an N-terminal domain which contains two EF-hand-like structures and a C-terminal dynein light chain-like (DLC-like) domain. Their functions remain obscure although one report demonstrated that one family member (Sm20.8) interacts with a dynein light chain as part of a larger complex [18]. Many trematode species express several, different members of this protein family. *S. mansoni* expresses 13 family members [19–23] and therefore it seems likely that a similar number will be present in other members of the genus. Some proteins from *Schistosoma japonicum* and *Schistosoma haematobium* have already been identified and characterised [24–27]. *F. hepatica* and *Fasciola gigantica* each express at least four, and family members have also been identified in both *Clonorchis sinensis* and *Opisthorchis viverrini* [28–34]. Their uniqueness to parasitic platyhelminthes and the likelihood that they are involved in signalling or regulatory processes makes them attractive potential drug targets. However, there is currently only limited information about their biochemical properties.

Members of this protein family from *Schistosoma* spp have been shown to elicit IgE-mediated immune responses in the host [23,26,27,35,36]. For this reason, they have been named the tegumental allergen-like (TAL) protein family and are considered promising targets for the development of vaccines against schistosome infection (for example, see Ref. [37]). The first three members of the family (SmTAL1, SmTAL2 and SmTAL3) were all discovered independently and given alternative names. Sm22.6 (SmTAL1) has been shown to bind to and inhibit thrombin [38]. The protein is soluble, but may be associated with membrane proteins [19]. Its calcium ion and drug binding properties have not been investigated. No calcium binding was observed with Sm21.7 (SmTAL2) despite the presence of at least one potentially functional EF-hand [20]. Similarly, blotting with radioactive calcium ions did not reveal any calcium binding by Sm20.8 (SmTAL3) [21].

Here, we investigated the biochemical properties of SmTAL1, SmTAL2 and SmTAL3 with particular reference to ion and drug binding. Our results demonstrate that the biochemical properties of these three TAL protein family members differ markedly.

## 2 Materials and methods

### 2.1 Molecular modelling

Initial homology models were generated using Phyre2 in the intensive mode [39] and then computationally solvated and energy minimised using Yasara [40]. Calcium ions were added into both EF-hands in the SmTAL1 model by aligning it with the calcium-bound form of the N-terminal domain of soy bean calmodulin isoform 1 (PDB 2RO8 [41]). A new version of the SmTAL1 pdb file which included the calcium ion(s) from the aligned protein was saved and this model was then re-minimised using Yasara. A calcium ion was added into the second EF-hand of SmTAL2 using the same procedure and the calcium-bound form of a protein-engineered calcium sensor (PDB 3U0K [42]) as a template. These proteins were chosen because they were highly ranked, calcium bound proteins used as templates in the homology modelling process. The final, minimised models in the apo and calcium-bound forms are presented as supplementary data to this paper.

### 2.2 Expression and purification of SmTAL1, SmTAL2 and SmTAL3

Recombinant SmTAL1 (AAA29922.1, Smp_045200.1), SmTAL2 (AAA74050.1, Smp_086480.1) and SmTAL3 (AAC79130.1, Smp_086530.1) were expressed in *Escherichia coli* as GST-fusion proteins (5′ GST) then isolated on Glutathione-agarose and cleaved with thrombin as previously described [23]. Free GST was removed by passing each thrombin digest through Q-Sepharose anion exchange beads (Amersham Bioscience) equilibrated with 50 mM Tris/HCl pH 8.0 containing 10 mM reduced glutathione. Contaminant thrombin was removed by addition of benzamadine-agarose beads (Sigma).

### 2.3 Native gel electrophoresis

All three SmTAL proteins were resolved in continuous, native gel electrophoresis. The different physical properties of the proteins meant that different conditions were required for each. SmTAL1 (17 μM), SmTAL2 (11 μM) or SmTAL3 (32 μM) was incubated at 20 °C for 30 min in the presence of EGTA (1 mM) or EGTA (1 mM)/cation (2 mM). An equal volume (10 μl) of native loading buffer was added (20% v/v of the appropriate running buffer, 20% v/v glycerol, 5% w/v bromophenol blue, 1% w/v DTT). SmTAL1 was electrophoresed on a 6% polyacrylamide gel at pH 8.8 (20 mA for 60 min on ice) with a running buffer containing 25 mM Tris–HCl, 250 mM glycine, pH 8.8. SmTAL2 was electrophoresed on a 6% polyacrylamide gel at pH 9.4 (20 mA for 90 min on ice) with a running buffer containing 60 mM Tris, 40 mM CAPS [43]. SmTAL3 was electrophoresed on 6% polyacrylamide gel at pH 7.4 (20 mA for 80 min on ice) with a running buffer containing 43 mM Imidazole, 35 mM Hepes [43]. Gels were stained with Coomassie blue and destained with 0.75% (v/v) acetic acid and 0.5% (v/v) ethanol.

### 2.4 Analytical methods

Differential scanning fluorimetry (DSF) was carried out using 5–7 μM protein in a total volume of 20 μl and the fluorescent dye Sypro Orange (10 × ; manufacturer's concentration definition) as previously described [44]. Divalent ions or drugs were added as appropriate. Drugs were initially dissolved in 100% DMSO and diluted in buffer R (50 mM Hepes-OH, pH 7.5, 150 mM NaCl, 10% v/v glycderol) as required. The concentration of DMSO never exceeded 1% v/v.

Limited proteolysis was carried out using 10–14 μM protein and 20–650 nM protease (trypsin, chymotrypsin or subtilisin) plus 0.8 mM calcium chloride. Reactions (10 μl) were incubated for approximately 10 min at 37 °C before addition of the protease. They were then incubated for a further 60 min before being stopped by the addition of an equal volume of SDS-loading buffer (120 mM TrisHCl pH 6.8, 4% (w/v) SDS, 20% (v/v) glycerol, 5% (w/v) bromophenol blue, 1% (w/v) DTT). Results were analysed by 15% SDS-PAGE.

Crosslinking with BS^3^ (50–500 μM) was carried out with 10–14 μM protein (diluted as required in buffer R) in a total volume of 10 μl. Reaction mixtures were incubated at 37 °C for 35 min before addition of the crosslinker and then incubated at the same temperature for a further 35 min. EGTA (0.8 mM) or calcium chloride (1.6 mM) was added as required. Reactions were stopped by the addition of an equal volume of SDS-loading buffer and analysed by 15% SDS-PAGE. Control reactions with recombinant human galactokinase (prepared as previously described [45]) were carried out using the same protocol.

Intrinsic fluorescence was measured using a Spectra Max Gemini XS fluorescence platereader fluorimeter and SOFTmax PRO software. Measurements were taken in triplicate in black 96-well plates. SmTAL proteins (7–8 μM) were diluted in 10 mM Hepes-OH, pH 8.8 and calcium chloride (0.8 mM) and/or EGTA (0.4 mM) were included as required. Fluorescence was excited at 280 nm and emission measured from 320 to 420 nm. Spectra were corrected by subtraction of the background spectrum resulting from the same volume of buffer supplemented with calcium chloride and/or EGTA as appropriate.

Protein concentrations were determined by the method of Bradford [46] using BSA as a standard.

## 3 Results and discussion

### 3.1 SmTAL proteins show differences in their predicted structures

Homology modelling of the three SmTAL proteins predicted that, as expected from protein sequence analysis, each protein consisted of an N-terminal, largely α-helical domain containing two EF-hand-like structures and a C-terminal, largely β-sheet domain (Supplementary Figure S1). It should be noted that the linker between the domains lacks secondary structure, is likely to be flexible and is unlikely to be predicted well by the methods used. Therefore, the orientation of the domains with respect to each other is likely to vary as a consequence of the linkers’ flexibilities. The C-terminal domains resemble the typical structure of a dynein light chain. Recently a preliminary report of the experimental structure of the C-terminal domain of SmTAL2 demonstrated that this protein adopts a DLC-like fold [47], supporting the predictions from our models. The overall structures of the SmTAL proteins are similar to those predicted for the *F. hepatica* proteins FhCaBP3 and FhCaBP4 [30,31].

Despite the similarity in domain organisation, there are some key differences in the structures. Both SmTAL1 and SmTAL3 form more extended structures, whereas SmTAL2 is more compact. The main cause of this difference is the length of the flexible, essentially unstructured linker between the two domains. In SmTAL1 it is 26 residues long (Ser-76 to Ile-100) and in SmTAL3 it spans 17 residues (Gln-75 to Ile-91). In contrast the linker in SmTAL2 is only five residues long (Gly-67 to Asn-71). Consequently, the folded part of the C-terminal domain in this protein is larger than in the other two proteins and it is also in closer proximity to the N-terminal domain.

Examination of the structure and sequences of the N-terminal domains revealed two potential EF-hand calcium binding sequences in each of the three proteins. A typical EF-hand is structured so that six residues can coordinate the calcium ion using a mixture of side chain and backbone oxygen atoms. The coordinating residues anchor the ion approximately at right angles to each other and are referred to as X, Y, Z, -X, -Y and -Z [17]. The EF-hand folds into a loop, with residues not involved in ion binding facilitating the tight turns necessary for this structure [48]. Bioinformatics analyses have revealed preferred residues at each of the coordinating positions [17].

**Fig. 1:**
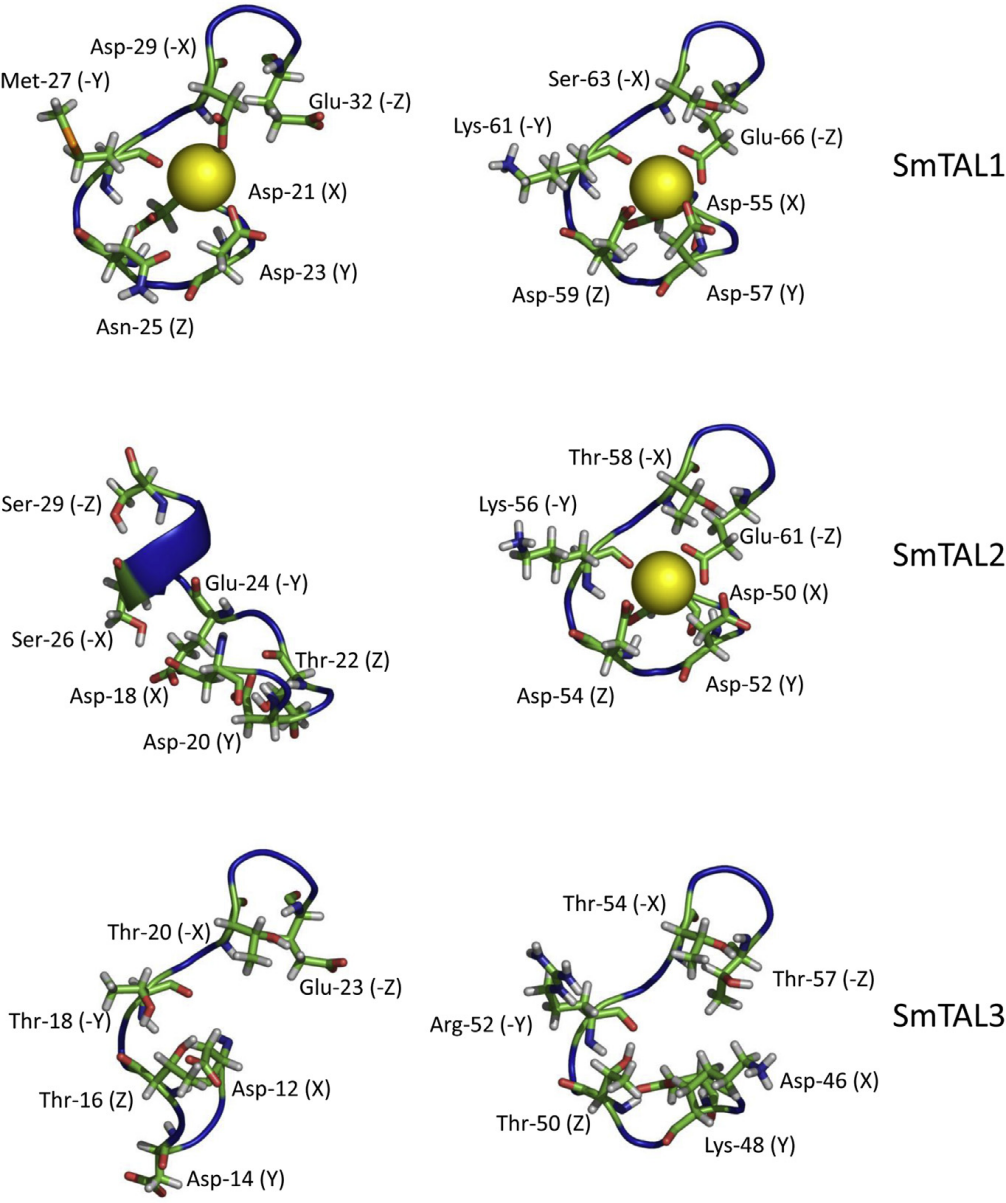
**EF-hands from the SmTAL proteins**. Molecular models of the EF-hand sequences from each of the SmTAL proteins are shown. In each case the potential ion coordinating residues are shown. For SmTAL1 both EF-hands are shown occupied by a calcium ion. In SmTAL2 only the second EF-hand is shown occupied and in SmTAL3 neither is shown occupied. These calcium ion occupancies are consistent with predictions based on the structure and sequences, and with the experimental data presented in this paper (see Results and Discussion).

In SmTAL1, both EF-hands are folded into the typical loop (Fig. 1). In the first motif, the potentially coordinating residues conform to the preferred ones, expect at -Y (Met-27, where threonine is preferred). However, this at this position coordination is provided by the backbone oxygen. In the model presented here, this oxygen atom is orientated into the potential ion binding space (Fig. 1). The second EF-hand in SmTAL1 also has the typical structure and this motif has preferred residues at all the potentially coordinating positions. Thus, based on these predictions, both EF-hands have the potential to be functional calcium binding sites (Fig. 1).

The first motif in SmTAL2 deviates from both the typical sequence and structure of an EF-hand. The Z residue (Thr-22, aspartate preferred) and -Y (Glu-24, threonine preferred) both deviate from the preferred residues. The predicted structure also differs from the typical EF-hand fold being elongated in comparison and partly folded into an α-helix (Fig. 1). It is, therefore, unlikely that this motif interacts with calcium ions. The second EF-hand in SmTAL2 conforms to the consensus fold and all the potentially coordinating residues are preferred ones. Therefore, this EF-hand is likely to bind calcium ions (Fig. 1).

Both EF-hands in SmTAL3 deviate considerably from the consensus. The first EF-hand adopts an elongated structure (similar to the first EF-hand in SmTAL2) and deviates from the preferred residue at positions Z (Thr-16) (Fig. 1). The second motif resembles the fold of a typical EF-hand. However, it differs from the preferred coordinating residues at Y (Lys-48), Z (Thr-50) and –Z (Thr-56). Of these, the lysine residue is probably the most significant. It introduces a positive charge into the motif (thus potentially repelling the cation) and removes one of the coordinating oxygens (Fig. 1). Therefore it is unlikely, based on this predicted structure, that either EF-hand in SmTAL3 will be functional as a calcium ion binding site.

### 3.2 SmTAL proteins have different divalent cation binding properties

The three SmTAL proteins were tested for their ability to bind calcium ions by native gel electrophoresis, intrinsic fluorescence spectroscopy and DSF. Each protein has different electrophoretic properties and it was necessary to use different gel systems for each protein. Based on previous experience with FhCaBP3 [31], the calcium chelating agent EGTA was routinely added to the SmTAL proteins to remove any calcium ions which bound during recombinant expression/purification. Divalent ions were then added in a two-fold molar excess as required.

**Fig. 2:**
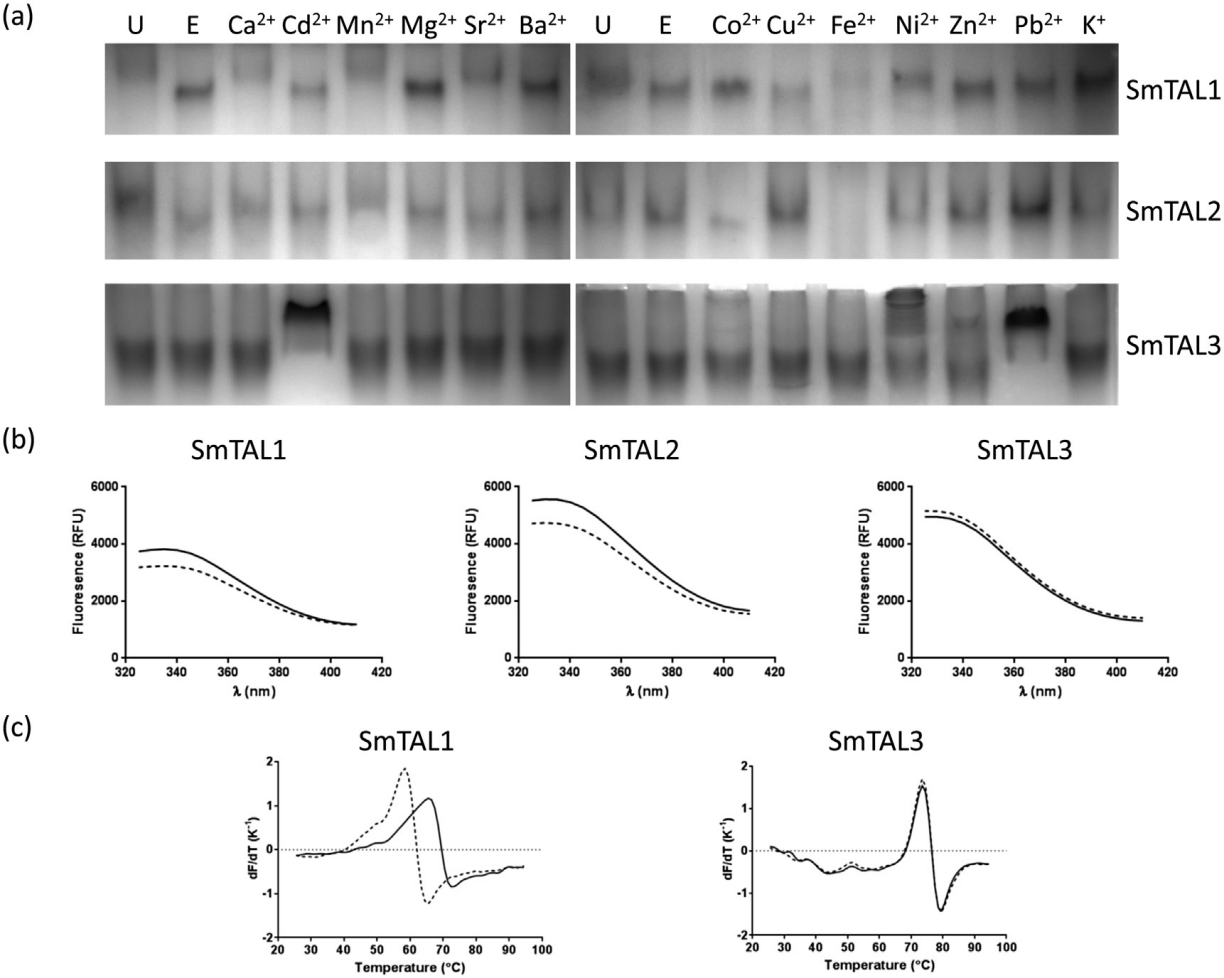
**Ion binding by SmTAL proteins**. (a) Native gel electrophoresis of SmTAL1 (17 μM), SmTAL2 (11 μM) and SmTAL3 (32 μM). U, untreated protein; E, protein plus 1 mM EGTA; various ions as indicated at a concentration of 2 mM ion/1 mM EGTA. For the conditions of electrophoresis see Materials and Methods. Given that the proteins were shown to be dimeric in crosslinking experiments, it is reasonable to assume that the bands in these native gels also represent dimers. (b) Intrinsic fluorescence spectra of SmTAL1 (7 μM), SmTAL2 (8 μM) and SmTAL3 (7 μM) in the 0.4 mM EGTA (dashed line) and 0.4 mM EGTA/0.8 mM calcium chloride (solid line). (c) First derivative curves for the thermal denaturation (“melting”) of SmTAL1 (5 μM) and SmTAL3 (7 μM). Dashed line, protein plus 0.4 mM EGTA; solid line, protein plus 0.4 mM EGTA/0.8 mM calcium chloride.

SmTAL1's electrophoretic mobility was increased in the presence of EGTA compared to the untreated protein; addition of calcium ions reduced the mobility to a level similar to that of the untreated protein (Fig. 2a). Thus, it is likely that the recombinant protein is largely calcium-bound and that SmTAL1 can reversibly bind to calcium ions. Similar shifts were also seen following the addition of manganese, strontium, nickel (II) and, possibly, iron (II) ions. However, cadmium, magnesium, barium, cobalt (II), copper (II), zinc, lead (II) and potassium ions did not result in any shift (Fig. 2a). SmTAL2 shows similar behaviour in the presence of EGTA or calcium ions (Fig. 2a). Therefore, it too was largely purified in a calcium-bound state and is capable of reversible binding to calcium ions. In addition to calcium ions, SmTAL2 also interacted with cadmium (II), manganese, magnesium and, possibly, strontium and barium ions (Fig. 2a). Iron (II) and cobalt (II) ions caused some blurring of the protein on the gel most likely as a result of protein aggregation or partial unfolding (Fig. 2a). With the exception of calcium and, perhaps, manganese, it is unlikely that any of the interactions with ions by SmTAL1 and SmTAL2 are physiologically relevant. Nevertheless, these results illustrate that, despite structurally similar EF-hands (Fig. 1), the two proteins show some variability in their ion binding properties. In contrast to SmTAL1 and SmTAL2, SmTAL3's mobility was unaffected by EGTA or by calcium ions. Indeed, none of the ions tested resulted in a shift similar to those seen with SmTAL1 or SmTAL2. Cadmium (II), nickel (II), zinc and lead (II) ions all caused blurring or a substantial shift suggesting aggregation or partial unfolding (Fig. 2a). These data suggest that SmTAL3 does not undergo a physiologically relevant, reversible interaction with any of the ions tested.

All three SmTAL proteins gave the expected fluorescence emission spectra when excited at 280 nm, ie a broad peak at approximately 340 nm (Fig. 2b). Spectra were recorded in 0.4 mM EGTA and in 0.4 mM EGTA/0.8 mM calcium chloride. In the case of SmTAL1 and SmTAL2, the spectra under these two conditions were noticeably different and the fluorescence intensity at the maximum wavelength was statistically significantly different (*p* < 0.02; unpaired *t*-test with Welch's correction). However, in the case of SmTAL3, there was no noticeable difference in the two spectra and no statistically significant difference in intensity at the peak emission wavelength (Fig. 2b). These data provide additional evidence that SmTAL1 and SmTAL2 are calcium ion binding proteins, whereas SmTAL3 is not.

All three SmTAL proteins showed considerable thermal stability (Table 1). In the presence of 0.4 mM EGTA (to remove any bound calcium ions), both SmTAL1 and SmTAL3 had melting temperatures well above the mammalian host body temperature. It was not possible to obtain reliable melting temperature data for SmTAL2. The DSF assay measures the increase in dye fluorescence when it is released from the unfolded protein [49]. With SmTAL2, no such increase was observed and so it was assumed that its melting temperature is higher than the limit of the instrument (95 °C). Both SmTAL1 and SmTAL3 showed a single phase thermal denaturation, suggesting that either the two domains have similar *T*_*m*_ values or that the proteins unfold in a single transition (which would imply that the domains contact each other in the native fold). SmTAL1, but not SmTAL3, was thermally stabilised by the presence of calcium ions (Fig. 2c; Table 1). Typically, proteins are stabilised by ligands which bind to the native state. Therefore, this result provides further evidence that SmTAL1 is a calcium binding protein whereas SmTAL3 is not. This experimental finding is consistent with the bioinformatics and homology modelling analyses which showed that both EF-hands in SmTAL3 deviated from the consensus (Fig. 1). Therefore, we conclude that SmTAL1 and SmTAL2 reversibly interact with calcium (and some other divalent ions) but SmTAL3 does not interact with calcium ions.

### 3.3 SmTAL proteins have different drug binding properties

The ability of the SmTAL proteins to interact with the calmodulin antagonists chlorpromazine (CPZ), N-(6-Aminohexyl)-5-chloro-1-naphthalenesulfonamide hydrochloride (W7) and trifluoperazine (TFP), the anti-schistosomal drug praziquantel and the barbiturate thiamylal was investigated by limited proteolysis and DSF. TFP, W7 and CPZ bind to partially overlapping hydrophobic pockets in the EF-hand domains of calmodulin [50–53]. Therefore, we hypothesised that they might interact with the SmTAL proteins through their EF-hand domains. PZQ has been shown to interact with myosin regulatory light chain (RLC) from *S. mansoni* [9]. The binding site has not been mapped. Myosin RLCs have similar folds to calmodulin and, therefore, we reasoned that PZQ might also interact with other proteins with similar, or partially similar, structures (such as SmTALs). Although thiamylal's main pharmacological action is as an anaesthetic, it has also been reported to disrupt the interaction between calmodulin and calcineurin, most likely through direct interaction with calmodulin [54]; however, the binding site on calmodulin has not been determined.

**Fig. 3:**
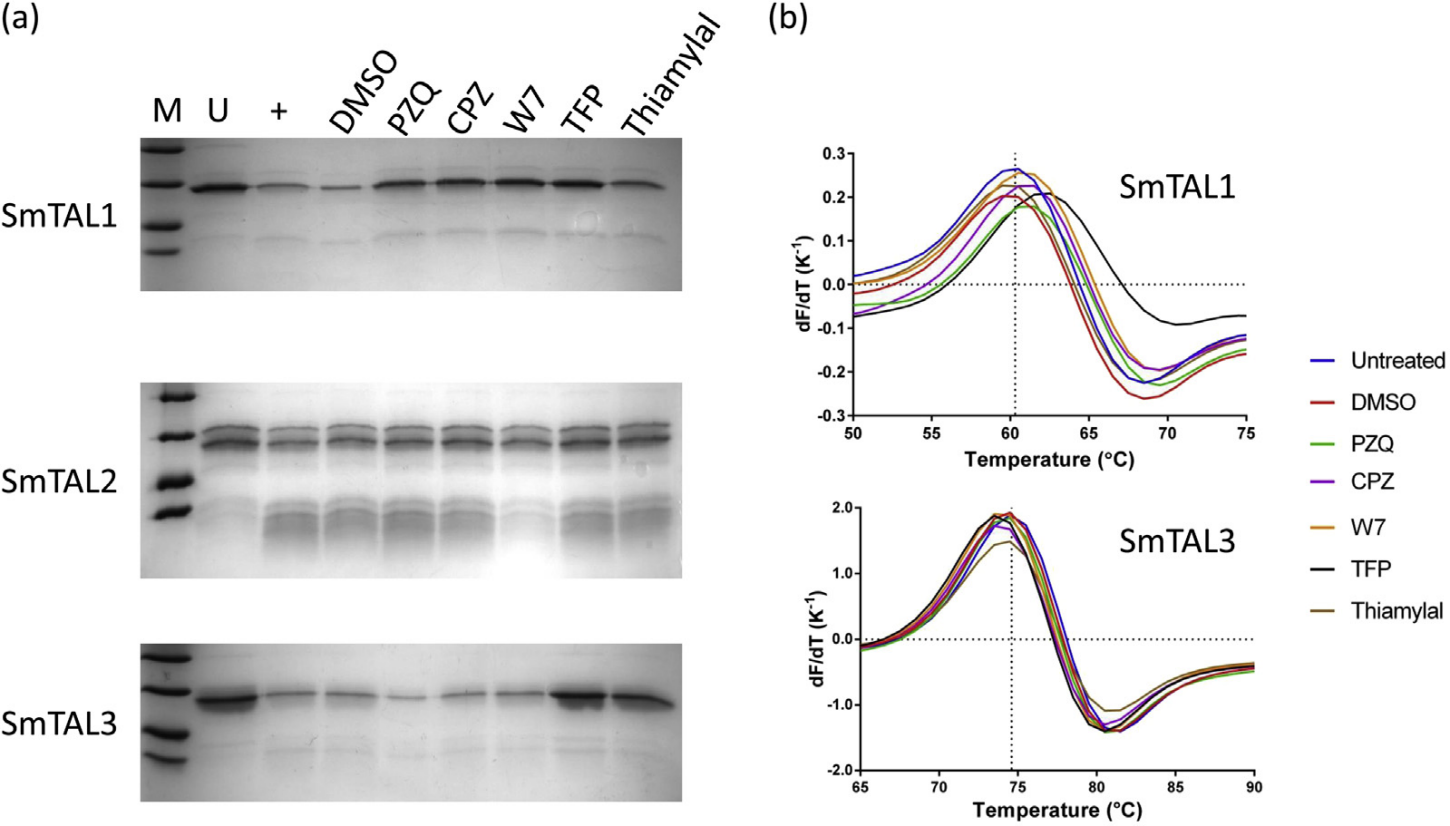
**Drug binding by SmTAL proteins**. (a) Limited proteolysis of SmTAL1 (14 μM), SmTAL2 (10 μM) and SmTAL3 (13 μM) with chymotrypsin (600 nM), trypsin (120 nM) and trypsin (650 nM) respectively. M, molecular mass markers (45, 35, 18, 14 kDa); U, untreated protein; +, protein in the presence of protease. The DMSO control and the reactions in the presence of drugs all contained 1% v/v DMSO and 0.8 mM calcium chloride. All drugs were present at a concentration of 250 μM and reactions were analysed by 15% SDS-PAGE. (b) First derivative curves for the thermal denaturation (“melting”) of SmTAL1 (5 μM) and SmTAL3 (7 μM) in the presence of drugs (each 250 μM in the presence of 1% v/v DMSO/0.8 mM calcium chloride). The vertical dotted lines on the graphs indicate the melting temperature of the protein in the presence of 1% v/v DMSO.

SmTAL1 was partially protected from limited proteolysis by chymotrypsin by PZQ, CPZ, W7, TFP and, possibly, thiamylal (Fig. 3). Similar results were observed with subtilisin (Supplementary Figure S2). This method has been previously used with calmodulin-like proteins from *F. hepatica* demonstrating that, as expected, TFP and W7 interacted with FhCaM1 (which differs from human calmodulin by just two amino acid residues) [55,56]. The same drugs, except thiamylal, caused a significant (*p* < 0.05; unpaired *t*-test with Welch's correction) increase in the melting temperature of the protein compared to the DMSO control (Fig. 3; Table 1). These data suggest that the drugs interact with SmTAL1 increasing its stability towards both proteolysis and thermal denaturation as a consequence. Of the drugs tested, only W7 protected SmTAL2 from limited proteolysis by trypsin or chymotrypsin (Fig. 3; Supplementary Figure S2). As noted above, it was not possible to carry out DSF analysis on this protein. Both TFP and thiamylal protected SmTAL3 from proteolysis by trypsin (Fig. 3). CPZ, TFP and W7 (but not PZQ or thiamylal) caused a significant (*p* < 0.05; unpaired *t*-test with Welch's correction) decrease in the melting temperature of SmTAL3 (Fig. 3; Table 1). A decrease in the melting temperature can arise from the drug binding to partially folded states and thus destabilising the overall population of protein molecules [57]. The situation with SmTAL3 is clearly more complex than with SmTAL1 where the results from limited proteolysis and DSF were broadly in agreement. However, the two techniques measure different consequences of ligand binding and a negative result does not provide evidence for a lack of a drug–protein interaction. Furthermore, it is possible for an interacting drug to show a positive result in only one of two experiments; indeed this has been observed previously with FhCaBP3 using limited proteolysis and fluorescence quenching [31]. While *T*_*m*_ values report on the protein's overall stability, proteolytic digestion reports on events in the immediate vicinity of the protease cleavage site. Therefore, it is not surprising to observe that TFP causes loss of overall stability as measured by DSF, while locally rigidifying the polypeptide backbone (or sterically hindering access to the protease) in limited proteolysis experiments.

### 3.4 SmTAL proteins are dimeric

**Fig. 4:**
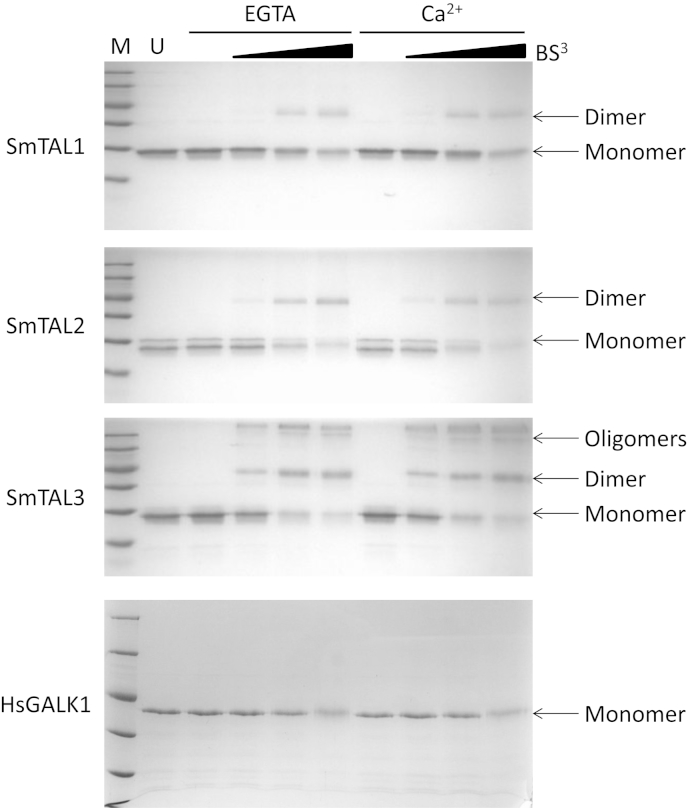
**Dimerisation of the SmTAL proteins**. The crosslinking of the SmTAL proteins by BS^3^ was monitored by 15% SDS-PAGE. M, molecular mass markers (116, 66, 45, 35, 25, 18 kDa); U, Untreated SmTAL protein (SmTAL1 and SmTAL3, 14 μM; SmTAL2, 10 μM); EGTA, reactions carried out in the presence of 0.8 mM EGTA; Ca^2+^, reactions carried out in 0.8 mM EGTA/1.6 mM calcium chloride; BS^3^ and associated triangle, increasing concentrations of the crosslinker (50, 150, 500 μM). As a negative control, human galactokinase (HsGALK1, 15 μM) was tested under the same conditions.

EF-hand containing proteins (e.g calmodulin, S100 family proteins) and dynein light chains can form homodimers [56,58–61]. Previously, it has been shown that FhCaBP3 forms homodimers, although the extent of dimerization is reduced in the presence of calcium ions [31]. In contrast, FhCaBP4 has a greater tendency to form dimers in the presence of calcium ions [30]. All three SmTAL proteins form dimers in solution as judged by protein–protein crosslinking and SmTAL3 forms higher order oligomers (Fig. 4). Calcium ions did not affect the extent of dimerization by any of the SmTAL proteins (Fig. 4). Control experiments with human galactokinase, which is known to be monomeric from analytical gel filtration experiments [62], showed no crosslinking under the same conditions (Fig. 4). This suggests that, in contrast to FhCaBP3 and FhCaBP4, calcium controlled dimerization does not form part of the mechanism of action of the SmTAL proteins and that all three proteins are likely to function as dimers *in vivo*.

### 3.5 Conclusions

Despite similarities in sequence and domain organisation, the three *S. mansoni* TAL proteins studied here have different biochemical properties. While SmTAL1 and SmTAL2 interact with calcium ions, the altered EF-hand sequences in SmTAL3 mean that it is unable to do so. The three proteins also have different drug binding properties: a range of compounds known to interfere with calcium signalling was tested and it was found that each protein interacted with a different subset of compounds. This demonstrated that is possible to distinguish between these proteins pharmacologically. Furthermore, these biochemical differences suggest that the proteins have different functions in the organism. Discovering the cellular roles of this family of proteins remains a major challenge in the field. To date, only one binding partner has been reported for these proteins: SmTAL3 interacts with a dynein light chain and, presumably, other unidentified proteins in a 90 kDa complex [18]. Whether, or not, other members of the SmTAL family can substitute for SmTAL3 in this complex is not known.

The discovery that SmTAL1 binds to praziquantel is interesting and it remains to be discovered if this interaction is pharmacologically important. Despite the successful use of this drug for over three decades, its molecular mechanism of action remains elusive. The majority of evidence points towards a mechanism which involves the disruption of calcium-mediated processes. The ability of SmTAL1 to bind calcium ions suggests the hypothesis that praziquantel acts, in part, through antagonism of this protein's activities. Calmodulin regulates some voltage-gated calcium channels in a variety of vertebrate and invertebrate species [63]. In some cases the channel is inactivated by calcium-bound calmodulin [64,65]. It is tempting to speculate that SmTAL1 (and possibly other members of this group of proteins) may be able to perform this function in Platyhelminthes. If this was the case, antagonism of SmTAL1 would result in dysregulation of the voltage-gated calcium channel and unregulated influx of calcium ions, in other words, the documented physiological consequences of PZQ in *Schistosoma* spp [66]. This merits further investigation.

## Conflict of interest

The authors have no conflicts of interest to declare.

## Figures and Tables

**Table 1:** Melting temperatures (in °C; means ± standard deviation) of SmTAL1 (5 μM) and SmTAL3 (7 μM) (determined by DSF) under various conditions.

	SmTAL1	SmTAL3
EGTA (0.4 mM)	58.4 ± 0.5	73.6 ± 0.4
EGTA (0.4 mM)/CaCl_2_ (0.8 mM)	65.7 ± 0.7^a^	73.6 ± 0.4
DMSO (1% v/v)	60.3 ± 0.1	74.6 ± 0.1
Praziquantel (0.25 mM)	61.7 ± 0.2^b^	74.5 ± 0.0
Chlorpromazine (0.25 mM)	61.3 ± 0.3^b^	74.1 ± 0.1^b^
W7 (0.25 mM)	61.5 ± 0.3^b^	74.3 ± 0.1^b^
Trifluoperazine (0.25 mM)	62.2 ± 0.8^b^	73.9 ± 0.1^b^
Thiamylal (0.25 mM)	60.5 ± 0.7	74.5 ± 0.4

a Significantly different (*p* < 0.05 in an unpaired *t*-test with Welch's correction) from the value for the same protein in EGTA only.

b Significantly different (*p* < 0.05 in an unpaired *t*-test with Welch's correction) from the value for the same protein in the presence of DMSO. In the experiments with drugs, the final concentration of DMSO was 1% v/v. The DMSO control and the experiments with drugs all contain 0.8 mM calcium chloride.
