# Supplementary material for: Comparative biochemical analysis of three members of the Schistosoma mansoni TAL family: Differences in ion and drug binding properties
Source: Biochimie. 2015 Jan;108:40–7. doi: 10.1016/j.biochi.2014.10.015 (PMC4300400; doi:10.1016/j.biochi.2014.10.015)
Supplement: Supplementary file 6 [file mmc6.pptx]

## Slide 1
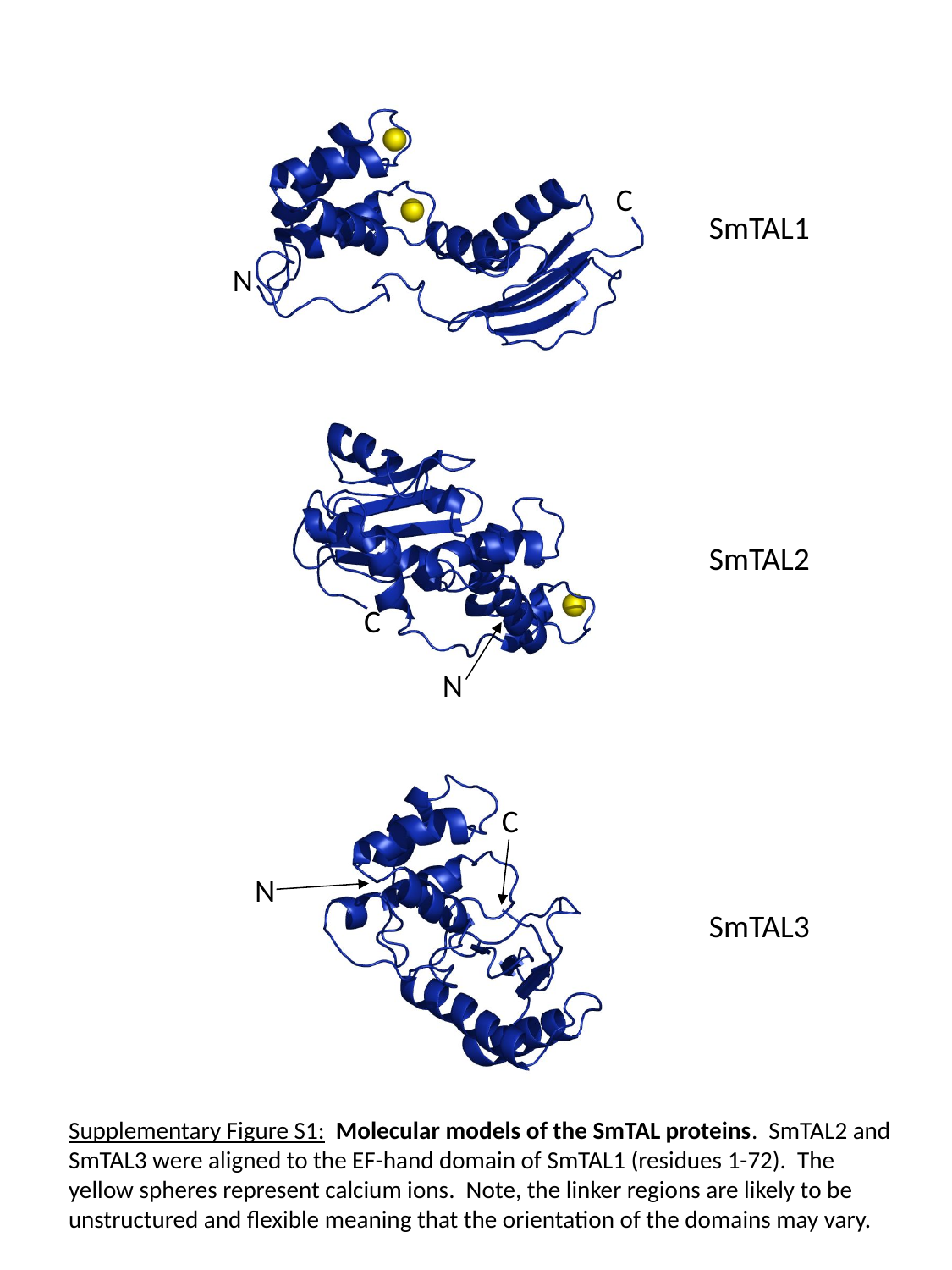

C
N
SmTAL1
C
N
SmTAL2
C
N
SmTAL3
Supplementary Figure S1: Molecular models of the SmTAL proteins. SmTAL2 and SmTAL3 were aligned to the EF-hand domain of SmTAL1 (residues 1-72). The yellow spheres represent calcium ions. Note, the linker regions are likely to be unstructured and flexible meaning that the orientation of the domains may vary.
