# Supplementary material for: Comparative biochemical analysis of three members of the Schistosoma mansoni TAL family: Differences in ion and drug binding properties
Source: Biochimie. 2015 Jan;108:40–7. doi: 10.1016/j.biochi.2014.10.015 (PMC4300400; doi:10.1016/j.biochi.2014.10.015)
Supplement: Supplementary file 7 [file mmc7.pptx]

## Slide 1
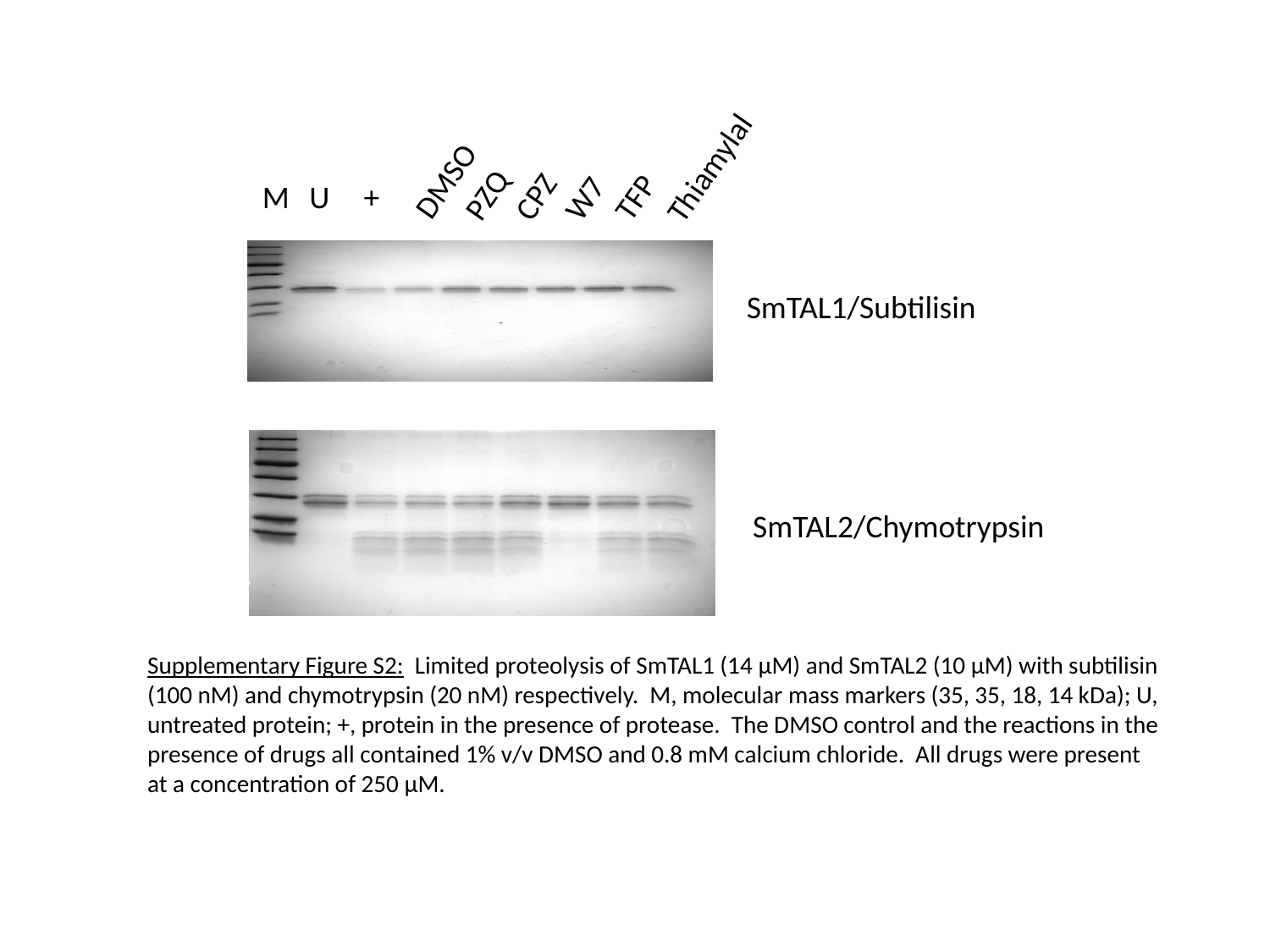

Thiamylal
DMSO
PZQ
CPZ
TFP
M
U
+
W7
SmTAL1/Subtilisin
SmTAL2/Chymotrypsin
Supplementary Figure S2: Limited proteolysis of SmTAL1 (14 μM) and SmTAL2 (10 μM) with subtilisin (100 nM) and chymotrypsin (20 nM) respectively. M, molecular mass markers (35, 35, 18, 14 kDa); U, untreated protein; +, protein in the presence of protease. The DMSO control and the reactions in the presence of drugs all contained 1% v/v DMSO and 0.8 mM calcium chloride. All drugs were present at a concentration of 250 μM.
